# **A new Helicoverpa armigera Nucleopolyhedrovirus isolate from **Heliothis peltigera** (Denis & Schiffermuller) (Lepidoptera: Noctuidae) in Turkey**

**DOI:** 10.3906/biy-1902-64

**Published:** 2019-10-14

**Authors:** Gözde Büşra EROĞLU, Remziye NALÇACIOĞLU, Zihni DEMİRBAĞ

**Affiliations:** 1 Department of Biology, Faculty of Science, Karadeniz Technical University, Trabzon, Turkey

**Keywords:** Safflower, baculovirus, nucleopolyhedrovirus, biocontrol, Heliothis peltigera

## Abstract

This study reports a new Helicoverpa armigera nucleopolyhedrovirus (NPV) isolated from Heliothis peltigera (Denis & Schiffermuller), collected in the vicinity of Adana, Turkey. Infection was confirmed by tissue polymerase chain reaction and sequence analysis. Results showed that dead H. peltigera larvae contain Helicoverpa armigera nucleopolyhedrovirus. Thus, the isolate was named as HearNPV-TR. Microscopy studies indicated that occlusion bodies were 0.73 to 1.66 μm in diameter. The nucleocapsids are approximately 184 × 41 nm in size. The genome of HearNPV-TR was digested with KpnI and XhoI enzymes and calculated as 130.5 kb. Phylogenetic analysis showed that HearNPV-TR has close relation with the H. armigera SNPV-1073 China isolate. The Kimura analysis confirmed that the isolate is a variant of H. armigera NPV. Bioassays were performed using six different concentrations (1 × 10^3^ to 1 × 10^8^ occlusion bodies (OBs)/mL) on 2nd instar larvae of H. peltigera, H. armigera, Heliothis viriplaca, Heliothis nubigera. LC_50_ values were calculated to be 9.5 × 10^3^, 1.9 × 10^4^, 8.6 × 10^4^ and 9.2 × 10^4^ OBs/mL within 14 days, respectively. Results showed that it is a promising biocontrol agent against Heliothinae species.

## 1. Introduction

The bordered straw, *Heliothis peltigera* (Denis & Schiffermuller) is one of the most serious pests infesting various economic crops such as safflower, cotton, soybean, corn, bean, and herbs (Manjunath et al., 1976; Iqbal and Mohyuddin, 1990; Saeidi and Adam, 2011). *H. peltigera* is distributed in the paleosubtropical regions (Hacker, 1989); however, as a result of climatic changes, such as global warming, it is currently becoming a major problem in the European countries (Ragionieri et al., 2017). Cultural methods, natural enemies, and some chemical agents are used in the control of *H. peltigera* (Simoglou et al.,**2017). The application of cultural control methods and natural enemies may be insufficient in epidemics of pest. Additionally, chemical pesticides damage nontarget organisms and environment, and it causes increase of insect resistance against chemicals (Ffrench-Constant et al., 2004). 

Baculoviruses are important biocontrol agents of insect pests in agriculture due to their narrow host range, persistence in the environment, being eco-friendly, and having high virulence (Fuxa, 2004; Wang et al., 2005). They are divided into two groups as nucleopolyhedrosis viruses (NPV) and granulosis viruses (GV) according to their polyhedrin and granule morphology (Ackermann and Smirnoff, 1983). Molecular classification of baculoviruses is based on the use of conserved gene regions of polyhedrine (*polh*), late expression factor 8 (*lef*8), and late expression factor 9 (*lef*9) (Jehle et al., 2006). 

Baculoviruses are used broadly as bio-insecticides to control Heliothinae**species in various vegetables and cotton (Ignoffo, 1973; Cunningham, 1998; Yang, 2012; Mironidis et al.,**2013). At present, there are many registered products belonging to Helicoverpa armigera and Helicoverpa zea nucleopolyhedroviruses in the world (Erlandson et al., 2007; Yang, 2012). They are both used against their own hosts and other Heliothinae**species (Beas-Catena et al., 2014). However, application of exotic virus isolates may cause adverse effects on native isolates (Munoz et al., 1997). Moreover, native isolates can be more pathogenic as per exotic isolates against local populations (Barrera et al., 2011; Cabodevilla et al., 2011). For these reasons, it is important to identify native isolates that indicate high virulence in each geographical region and use them against native pests (Figueiredo et al., 2009). 

In this study, a novel H. armigera NPV isolate was recorded the first time in *H. peltigera* host collected from safflower fields in Adana, Turkey. So far, no record of microbial control agent has been mentioned from this pest. Morphological and molecular features of this isolate were determined and insecticidal activity tests, using various concentrations, were performed against all larval stages of four Heliothinae**species (*H. peltigera*, armigera, *Heliothis viriplaca*, *Heliothis nubigera*) distributed in Turkey. Results showed that HearNPV-TR isolate is a promising biocontrol agent that could be used to control Heliothinae species.

## 2. Materials and methods

### 2.1. Virus detection and propagation

In July 2014, infestations of safflower crops by *H. peltigera* were observed in the vicinity of Adana, Turkey. Symptoms of larval feeding damage were observed on capitulum of safflower plants. A total of 357 larvae were collected from the field and brought to the laboratory. During laboratory rearing, five larvae died in a short time with typical baculovirus infection signs. Dead larvae were checked for the presence of baculovirus occlusion bodies (OBs) under light microscope (Nikon eclipse E600). 

Tissue PCR was performed to confirm the presence of baculovirus infections molecularly. Dead larvae were homogenized in sterile water and filtered through cheesecloth to remove the larval debris. The filtered OBs were purified as described by . OBs were dissolved using 0.1 M NaOH (pH 12.5) and subsequently, neutralized with 100 mM Tris-HCl (pH 8). The Phire Animal Tissue Direct PCR Kit (Thermo Fisher Scientific, Lithuania) was used to amplify the conserved regions of the baculovirus *lef*8, *lef*9, and *polh* genes. These genes were identified in all completely sequenced baculovirus genomes. Thus, they were detected in baculovirus phylogeny analysis (Jehle et al., 2006). The degenerate primer sets of these genes are shown in Table 1. Tissue-PCR reaction mixture was prepared following the manufacturer’s standard protocol for animal tissues. Amplified PCR products were sequenced by Macrogen Company (the Netherlands). Sequences were first blasted for alignment of the baculoviruses in NCBI (The National Center for Biotechnology Information) web site, subsequently registered to the GenBank.

**Table 1:** Primers of conserved gene regions used for phylogenetic analysis.

Insect	Intercept	Slope ± SE	LC_50_ (FL, 95%)	χ2	df
H. peltigera	–2.0 ± 0.3	0.40 ± 0.06	9.5 × 103 (8.9 × 103 – 1.02 × 104)	1.6	4
H. armigera	–1.9 ± 0.3	0.45 ± 0.06	1.9 × 104 (1.3 × 104 – 2.7 × 104)	1.8	4
H. viriplaca	–1.7 ± 0.3	0.50 ± 0.08	8.6 × 104(7.8 × 103 – 9.4 × 104)	2.0	5
H. nubigera	–1.6 ± 0.3	0.55 ± 0.08	9.2 × 104(8.7 × 103 – 9.8 × 104)	2.2	5

The virus was propagated freshly in the laboratory in healthy *H. peltigera *larvae. Insects were infected individually with diet (Poitout and Bues, 1974) discs contaminated with 10 µL virus suspension in a concentration of 10^5^ OBs/mL and maintained at 25 °C to develop infection. The OBs were purified from infected larvae as described by Ishii et al. (2003) and stored at –20 °C. 

### 2.2. Electron microscopy studies

OB morphology was studied under scanning electron microscope and transmission electron microscope. Twenty-five microliters of the OB suspension was spread on a stub and dried at 37 °C for 16 h. The stub was covered with gold dust (Quorum Technology SC7620-CF) and examined to measure the sizes of OBs (Agilent Technologies). 

For transmission electron microscopic examination, previously isolated OBs were washed twice with PBS and precipitated by high-speed centrifugation. The precipitated OB pellet was fixed in a fixative containing 2% glutaraldehyde, 2% paraformaldehyde in a 0.05 M pH 7.2 cacodylate buffer + 0.001 M CaCl_2_ for 2 h, postfixed in 1% OsO_4_ in the same fixative for 1 h, and subsequently embedded in resin. Ultrathin sections were obtained from OB blocks embedded in resin using an Ultracut S Ultramicrotome (Reichert, Munich, Germany). The sections, stained in 1% uranyl acetate and 1% lead citrate, were examined with a transmission (JEOL JEM 1220) electron microscope.

### 2.3. DNA extraction and restriction endonuclease profile

Viral DNA was extracted according to the method described by Reed et al. (2003) and dialyzed for 24 h at 4 °C against a 0.1 × TE buffer (10mM Tris/HCl, 1mM EDTA, pH 7.5) following phenol:chloroform:isoamyl alcohol extraction. The buffer was changed every 12 h. The extracted DNA was quantified using a spectrophotometer (Thermo Scientific, NanoDrop 2000). 

To determine the restriction profile of viral genome, 2 µg of DNA was digested with KpnI and XhoI enzymes for 4 h at 37 °C. The digested fragments were separated in 0.6% agarose gel at 15 V for 16 h. The agarose gel was then stained with ethidium bromide for 15 min and analyzed under UV light.

### 2.4. Phylogeny and Kimura-2 parameter analysis

In order to show the position of the HearNPV-TR among the other Heliothinae NPVs in GenBank, Kimura-2 parameter analysis was performed. For this reason, *polh*, *lef*8, and *lef*9 gene sequences of all isolates were concatenated to obtain a single phylogenetic tree of these genes. The nucleotide sequences were assembled and edited using BioEdit version 7.0.5.3 (Hall, 1999). The MEGA program (version 6.0) and the maximum composite likelihood method with 1000 bootstrap replicates were used for phylogenetic analysis. H. armigera granulosis virus isolate was used as an outgroup. 

Distance matrices between HearNPV-TR and other Heliothinae species were determined for partial *lef*8, *lef*9, and *polh* sequences using the pairwise distance calculation of MEGA version 6.0 applying the Kimura-2 parameter model. H. armigera granulosis virus, Trichoplusia ni nucleopolyhedrovirus, and Pseudoplusia includens nucleopolyhedrovirus were also included in the Kimura-2 parameter analysis. 

### 2.5. Biological tests

*H. peltigera* and H. armigera larvae were collected from safflower and sunflower fields, respectively, in Çukurova Region of Turkey. Heliothis viriplaca and Heliothis nubigera larvae, which are legume pests, were collected from the chickpea and lentil fields in the Southeastern Anatolia Region of Turkey, respectively. A generation of each larvae were reared to use for insecticidal tests and to determine the host range of HearNPV-TR in a climate cabinet at 26 °C and 65% humidity. Six different virus concentrations (1 × 10^3^, 1 × 10^4^, 1 × 10^5^, 1 × 10^6^, 1 × 10^7^, 1 × 10^8^ OBs/mL–1) were used for biological tests. Second instar of *H. peltigera*, H. armigera, *H. viriplaca*, and *H. nubigera* larvae were infected with virus contaminated semisynthetic diets. For the control group, sterile water was applied on diet. Thirty larvae were used for each test and the experiments were repeated 3 times. Mortality was assessed daily for 14 days. The mortality rates were determined by Abbott’s formula (Abbott, 1925), and the LC_50_ values were determined using probit regression analysis in SPSS (24.0 version). 

## 3. Results 

### 3.1. Virus detection and microscopy

Light microscopy examinations of dead *H. peltigera* larvae, collected from safflower fields in Adana, Turkey, showed typical baculovirus OBs as bright crystals. To verify baculovirus infection, partial sequences of *lef*8, *lef*9, and *polh* genes were amplified by tissue PCR. Amplified products of the expected sizes (*lef*8: 800 bp, *lef*9: 350 bp, and *polh*: 420 bp) were sequenced. Blast analysis of sequence results showed that deaths of the* H. peltigera* larvae were due to H. armigera nucleopolyhedrovirus infection. Thus, we have designated the isolate as HearNPV-TR. The sequences were registered to the GenBank and the accession numbers for *lef*8 (MG870624), *lef*9 (MG870625), and *polh* (MH161372) genes were obtained.

**Figure 1:**
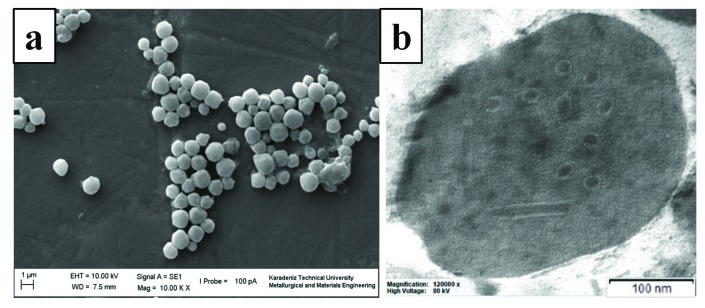
Micrographs of HearNPV-TR isolate. (a) Scanning electron micrographs of purified OBs, (b) Transmission electron micrographs of cross section of OBs.

Scanning electron microscopy studies showed that OBs were irregularly shaped and ranged in size from 0.73 to 1.66 μm (Figure 1a). Transmission electron microscopy examinations of HearNPV-TR OBs demonstrated several virions with a single nucleocapsid packaged within a single viral envelope and confirmed that HearNPV-TR was a single nucleocapsid-type virus (SNPV). The dimensions of rod-shaped nucleocapsids were approximately 184 × 41 nm (Figure 1b).

### 3.2. Restriction endonuclease profile

**Figure 2:**
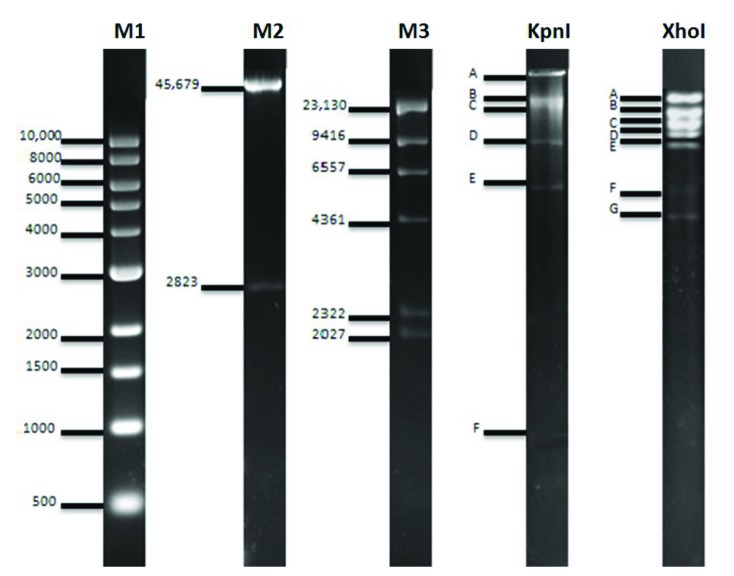
Restriction endonuclease profiles of HearNPV-TR digested with KpnI and XhoI enzymes. M1: 1 kb Marker (bp); M2: λ DNA/NarI; M3: λ DNA/HindIII.

The DNA of the HearNPV-TR, digested with KpnI and XhoI restriction enzymes, yielded six and seven fragments, respectively. These fragments were designated from A to G on Figure 2. Lambda (λ) DNA, digested with NarI and HindIII enzymes, was used as a molecular size marker. The sizes of the fragments relative to λ DNA/NarI and λ DNA/HindIII fragments are given in Table 2. Genome size of HearNPV-TR was calculated to be nearly 130.5 kbp.

**Table 2:** Restriction fragment and total genome sizes of HearNPVTR digested with KpnI and XhoI restriction endonucleases.

Fragment	KpnI	XhoI
A	54.8	41.8
B	34.5	35.6
C	24.8	19.9
D	9.5	13.1
E	6.0	11.2
F	0.9	4.5
G	-	4.4
Total	130.5 kb	130.5 kb

### 3.3. Phylogeny and Kimura-2 parameter analysis

**Figure 3:**
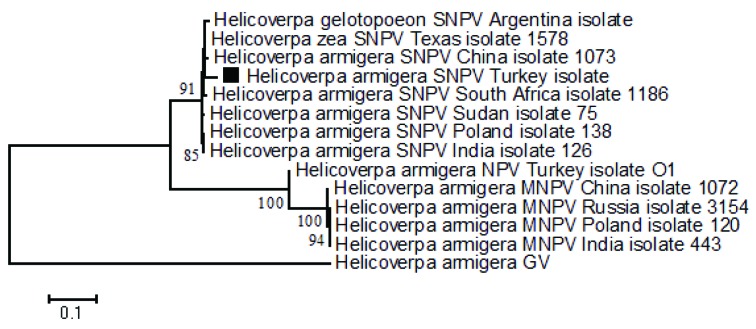
Neighbor joining trees based on concatenated partial sequences of lef8, lef9, and polh genes. It shows the phylogenetic position of HearNPV-TR between the other Heliothinae NPV isolates. The numbers indicate the bootstrap scores.

Phylogenetic analysis was performed to indicate the relationship of HearNPV-TR isolated from *H. peltigera *with the other Heliothinae NPVs (Table 3). The phylogenetic tree showed close relation of HearNPV-TR with H. armigera SNPV-1073 China isolate (Figure 3).

**Table 3:** Sequences information of baculoviruses isolates in phylogenetic tree.

Viral isolates	Origin	NCBI no lef8 lef9 polh		
Helicoverpa armigera SNPV-TR	Turkey	MG870624	MG870625	MH161372
Helicoverpa armigera MNPV -3154	Russia	HQ246047	HQ246039	HQ246031
Helicoverpa armigera MNPV -120	Poland	HQ246040	HQ246032	HQ246024
Helicoverpa armigera MNPV -1072	China	HQ246044	HQ246036	HQ246028
Helicoverpa armigera MNPV -443	India	HQ246042	HQ246034	HQ246026
Helicoverpa gelotopoeon SNPV	Argentina	KP340515	KP340516	KP340517
Helicoverpa zea SNPV-1578	Texas	HQ246115	HQ246142	HQ246088
Helicoverpa armigera SNPV -1073	China	HQ246108	HQ246135	HQ246081
Helicoverpa armigera SNPV -138	Poland	HQ246100	HQ246127	HQ246073
Helicoverpa armigera NPV -O1	Turkey	MH161371	MH161372	MG870623
Helicoverpa armigera SNPV -126	India	HQ246099	HQ246126	HQ246072
Helicoverpa armigera SNPV-75	Sudan	HQ246098	HQ246125	HQ246071
Helicoverpa armigera SNPV -1186	South Africa	HQ246112	HQ246139	HQ246085
Helicoverpa armigera GV	USA	EU255577	EU255577	EU255577

Kimura-2 parameter analysis of nucleotide distances indicated that the viral isolate in this study is a H. armigera NPV isolate. If the value of the nucleotide locus distances is less than 0.015, two baculoviruses are considered to be the same species and more than 0.050 are considered to be different species (Jehle et al., 2006). Distances between HearNPV-TR and other HearNPV isolates in GenBank were less than 0.015. However, distances between H. armigera GV and the other NPVs were greater than 0.050 (Table 4).

**Table 4:** Kimura-2 parameter analysis of HearNPV-TR.

polh/lef8/lef9		1	2	3	4	5	6	7	8	9	10	11	12	13	14	15
1	HearNPV-TR															
2	HearMNPV-3154	0.011														
3	HearMNPV-120	0.010	0.009													
4	HearMNPV-1072	0.013	0.002	0.008												
5	HearMNPV-443	0.013	0.007	0.002	0.007											
6	HegeSNPV	0.012	0.005	0.010	0.006	0.009										
7	HzSNPV-1578	0.009	0.006	0.010	0.007	0.010	0.004									
8	HearSNPV-1073	0.007	0.009	0.001	0.008	0.004	0.010	0.010								
9	HearSNPV-138	0.004	0.001	0.009	0.002	0.007	0.005	0.007	0.009							
10	HearSNPV-O1	0.004	0.000	0.009	0.002	0.007	0.005	0.006	0.009	0.000						
11	HearSNPV-126	0.004	0.000	0.009	0.002	0.007	0.005	0.006	0.009	0.000	0.000					
12	HearSNPV-75	0.006	0.004	0.009	0.005	0.008	0.003	0.006	0.009	0.004	0.004	0.004				
13	HearSNPV-1186	0.007	0.005	0.009	0.005	0.009	0.000	0.004	0.009	0.005	0.005	0.005	0.003			
14	HaGV	0.608	0.608	0.608	0.607	0.607	0.608	0.608	0.608	0.608	0.607	0.607	0.607	0.610		
15	TnSNPV	0.378	0.380	0.380	0.381	0.381	0.380	0.380	0.381	0.380	0.380	0.380	0.585	0.382	0.382	
16	PsinSNPV-IE	0.380	0.381	0.380	0.382	0.383	0.381	0.381	0.383	0.381	0.381	0.381	0.595	0.382	0.380	0.123

### 3.4. Biological tests

**Figure 4:**
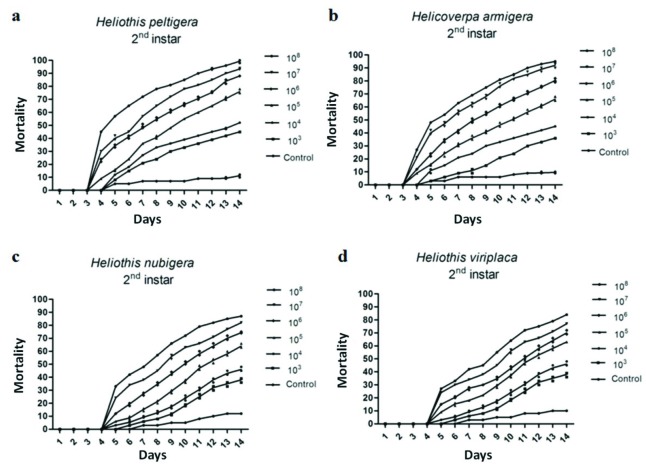
Virulence of HearNPV-TR isolate on 2nd instar larvae of H. peltigera (a), H. armigera (b), H. viriplaca (c), and H. nubigera (d).

To determine virulence of the HearNPV-TR isolate, biological tests have been carried out on both its host (*H. peltigera*) and other three Heliothinae**species (H. armigera, *H. viriplaca*, *H. nubigera*) distributed in Turkey. The LC_50_ of HearNPV-TR against the 2nd instar larvae of the *H. peltigera*,**H. armigera, *H. viriplaca*, and *H. nubigera* were determined to be 9.5 × 10^3^, 1.9 × 10^4^, 8.6 × 10^4^, and 9.2 × 10^4^ OBs/mL, respectively for 14 days (Figure 4, Table 5)

**Table 5:** Probit regression analysis values of HearNPV-TR isolate against 2nd instar larvae of H. peltigera, H. armigera, H. viriplaca, and H. nubigera.

Primer	Sequence	Size	Reference
lef8Flef8R	5’GTAAAACGACGGCCAGTTYTTYCAYGGNGA3’5’AACAGCTATGACCATGGNAYRTANGGRTCY3’	800 bp	Herniou, 2003
lef9Flef9R	5’CAGGAAACAGCTATGACCAARAAYGGITAYGCBG3’5’TGTAAAACGACGGCCAGTTTGTCDCCRTCRCARTC3’	350 bp	Lange et al., 2004
polhFpolhR	5’TAYGTGTAYGAYAACAAGT3’5’TTGTARAAGTTYTCCCAG3’	420 bp	de Moraes and Maruniak, 1997

SE: standard error, FL: fiducial limit, χ2: chi-square, df: degree of freedom.

## 4. Discussion

In this study, we detected a nucleopolyhedrovirus infection in *H. peltigera* larvae. As baculovirus isolates were named according to the host from which they were isolated, the name of this viral isolate was originally thought to be *H. peltigera* NPV. However, sequence results, phylogenetic analysis, and Kimura-2 parameter test showed that the isolate obtained from *H. peltigera* is a H. armigera single nucleopolyhedrovirus isolate. We previously reported another H. armigera NPV isolate from H. armigera larvae (Eroglu et al., 2018). However, the previous one was determined as a multinucleopolyhedrovirus. Therefore, the current virus isolate is totally different than the previous isolate. 

Electron microscopy studies are important for the morphological identification of baculovirus isolates. Scanning electron microscope results indicated HearNPV-TR OBs have sizes between 0.73 and 1.66 μm. When these OB sizes were compared to the OB sizes of other Heliothinae NPV isolates in the literature, it was obvious that HearNPV-TR has OB sizes smaller than those of Indian isolates (Somasekar et al., 1993) and China isolate (Tang et al., 2012). Indian isolates have OB sizes changing between 1.6 and 2.4 mm. China isolate has an OB size of approximately 2 mm. However, OB sizes of HearNPV-TR is larger than the OB size of Argentinian isolate (Ferrelli et al., 2016) which is between 0.6 and 1.2 mm. On the other hand, previous Turkish isolate, H. armigera NPV-O1, has OB sizes between 0.85 and 1.25 μm (Eroglu et al., 2018). 

TEM results revealed that HearNPV-TR has nucleocapsid sizes of 184 nm in length and 41 nm in width. Nucleocapsid sizes were also compared with the other Heliothinae NPV isolates in the literature. This comparison showed that HearNPV-TR has smaller nucleocapsid size than those of India isolate (Sridhar et al., 2011), China isolate (Tang et al., 2012), and previous Turkish isolate (Eroglu et al., 2018) which have nucleocapsid lengths and widths of 277 × 41 nm, 230 × 50 nm, and 279 × 56 nm, respectively. These results showed that the nucleocapsid size of HearNPV-TR isolate is significantly smaller than those of the other HearNPV isolates. This result suggested that nucleocapsid sizes varied between isolates obtained from different geographical regions.

HearNPV-TR was further characterized by restriction endonuclease digestion of the viral DNA. The size of the genome digested with KpnI and XhoI enzymes calculated to be approximately 130.5 kbp. This size is in accordance with the size of the viral genomes reported for *Helicoverpa* isolates in GenBank. Digestion with the same restriction enzymes was also reported for HearNPV China isolate (Chen et al., 2000). China isolate has similar restriction profile with HearNPV-TR for KpnI digestion, but digestion with XhoI enzyme produced 7 fragments for HearNPV -TR, 9 fragments for HearNPV China isolate. However, the genome size of HearNPV China isolate (130.1 kb) is almost at the same size with HearNPV-TR genome (130.5 kb). 

To see the position of HearNPV-TR between the other Heliothinae species in the GenBank, a phylogenetic tree was constructed using concatenated sequences of partial *lef*8, *lef*9, and *polh* genes. In the tree, HearNPV-TR was located near H. armigera SNPV-1073 China isolate (Rowley et al., 2011). Studies performed in the literature showed that Helicoverpa NPVs obtained from different hosts of *Helicoverpa* species are actually reported to be variants of each other (Jehle et al., 2006; Rowley et al., 2011; Wennmann et al., 2018). Thus, we concluded that HearNPV-TR was a H. armigera NPV variant. This result was also confirmed by the result of Kimura-2 parameter analysis performed by concatenated sequences of conserved partial genes (*lef*8, *lef*9, and *polh*). 

The LC_50_ values of these Heliothinae species were obtained as 9.5 × 10^3^, 1.9 × 10^4^, 8.6 × 10^4^, and 9.2 × 10^4^ OBs/mL, respectively. Kumar et al. (2011) also mentioned a similar result for their Indian HearNPV isolate which had produced a LC_50_ value of 2.3 × 10^4^ OBs/mL against the 2nd instar H. armigera larva. Furthermore we have found that HearNPV-TR is highly effective against all Heliothinae species found in Turkey (*H. peltigera*, *H. viriplaca*,**and *H. nubigera*). According to these results, it is possible to conclude that HearNPV-TR isolate is an encouraging biological control agent against Heliothinae species. 
